# Contrast associated nephropathy after intravenous administration: what is the magnitude of the problem?

**DOI:** 10.1080/0886022X.2021.1978490

**Published:** 2021-09-22

**Authors:** Jean-Sebastien Rachoin, Yanika Wolfe, Sharad Patel, Elizabeth Cerceo

**Affiliations:** a Department of Critical Care Medicine, Cooper University Health Care, Camden, NJ, USA; b Division of Hospital Medicine, Cooper University Health Care, Camden, NJ, USA

**Keywords:** Acute kidney injury, contrast-induced nephropathy, CT scan, outcomes, incidence, ESRD

## Abstract

Intravenous contrast media (CM) is often used in clinical practice to enhance CT scan imaging. For many years, contrast-induced nephropathy (CIN) was thought to be a common occurrence and to result in dire consequences. When treating patients with abnormal renal function, it is not unusual that clinicians postpone, cancel, or replace contrast-enhanced imaging with other, perhaps less informative tests. New studies however have challenged this paradigm and the true risk attributable to intravenous CM for the occurrence of CIN has become debatable. In this article, we review the latest relevant medical literature and aim to provide an evidence-based answer to questions surrounding the risk, outcomes, and potential mitigation strategies of CIN after intravenous CM administration.

## Introduction

Acute kidney injury in the setting of contrast medium (CM) administration has been extensively studied after it was first described in 1954 [1]. Since then, significant research has been devoted to the recognition, diagnosis, and prevention of this entity. Most of the earlier studies have focused on the consequences of intra-arterial (IA) CM administration in general and cardiac angiography in particular. It was not until 1985 that researchers analyzed the renal function of patients after CM-enhanced computed-tomography (CT) scans [2].

Much of this initial work was performed with high-osmolality CM which carried a greater risk of nephrotoxicity than the newer agents currently in use [3]. This resulted in the pervasive belief that the incidence of renal failure after CM was very elevated [4]. Furthermore, conclusions from the older studies on IA CM administration have been extrapolated to intravenous (IV) administration. Many clinicians still consider that both types result in similar rates of adverse events. It is not uncommon in daily clinical practice to have CM-enhanced studies be postponed, canceled, or replaced by lesser performing tests [such as ventilation-perfusion (V/Q) scan to diagnose pulmonary embolism], when patients have an abnormal renal function.

Over the last two decades, however, new research has challenged this paradigm. Recent studies surrounding IV administration of CM reported a much lower nephrotoxic risk than has been commonly cited in the past, especially in patients without significantly impaired renal function [5–8]. Some have suggested that renal failure after intravenous CM had been exaggerated and its true incidence and impact are more limited than initially thought [9].

In practice, this important issue needs careful consideration. On the one hand, if the administration of IV CM carries a significant risk in some (or all) patients, then we should use it judiciously, try to mitigate its consequences, and perhaps avoid it in certain cases. On the other hand, if the risks are not high and consequences are not dire, then life-saving tests and procedures should not be withheld or delayed. In 2020, *the American College of Radiology* and *the National Kidney Foundation (ACR/NKF)* published a consensus statement in which they downgraded the caution level related to IV CM administration [10]. Their conclusions are sound, balanced, and responsible and should be used as guidance in clinical practice.

In this article, we review the latest relevant literature and aim to provide an evidence-based answer to the following questions: What is the true incidence of contrast nephropathy after intravenous CM administration? Are there specific risk factors? Is there a difference with IA administration? Is there any way we can mitigate that risk? What are the consequences of this entity? We hope this will help clinicians make more informed decisions about the appropriateness of performing, postponing, or canceling contrast studies in their daily practices.

To do this we performed a systematic review of peer-reviewed articles pertaining to the incidence and impact of IV CM administration published in the English literature since 2010. The relevant study characteristics and findings are summarized in Table 1.

**Table 1.** Incidence of AKI after intravenous CM in studies from 2010 to 2021.

Study/year	Setting	Design	Patients	Definition CIN	Incidence CM group (%)	Incidence control (%)	Difference in AKI rates	Mortality (CM *vs.* control)	CKD or dialysis (CM *vs.* control)
Williams 2020 [55]	ICU	Propensity matched	4612	A1, B1	19.3	18	No difference		In hospital dialysis 0.43 *vs.* 0.25%
Hsu 2019 [48]	ED patients with sepsis	Propensity matched	587	B3	11.9	8.9	No difference	30-day mortality 25.7 *vs.* 27.7%	In hospital dialysis 8.9 *vs.* 6.9%
Goto 2019 [15]	ICU patient with Sepsis and AKI	Propensity matched	200	D	34	35	No difference	28-day mortality 9.2 *vs.* 15% 90 day mortality 25.8 *vs.* 32.1%	In hospital dialysis 26 *vs.* 23% Dialysis after discharge 0 *vs.* 2%
Miyamoto 2019 [49]	ICU patient with sepsis, and AKI requiring continuous dialysis	Propensity matched	6970					In hospital mortality 45.3 *vs.* 46.1%	Dialysis after discharge 4.4 *vs.* 4.1%
Hinson 2019 [21]	ED patients with sepsis	Propensity matched	4171	A3	7.2	9.6	No difference		
Hinson 2017 [20]	ED patients admitted to the hospital	Propensity matched	12 000	B3	10.6	10.2	No difference		CKD at 6 months 2 *vs.* 4.6% Dialysis at 6 months 0.4 *vs.* 0.9%
				A3	6.8	8.9	Higher in control group		
McDonald/2017 [57]**	ICU	Propensity matched	3016	A2	16.8	15.9	No difference	30 day mortality 21 *vs.* 17%	Dialysis at 7 days: 6.7 *vs.* 2.5%* (all with baseline GFR < 45 ml/min)
				B2	34.9	34.9			
Heller 2016 [50]	ED	Retrospective	7863	B6	8.6	9.6	No difference	In hospital mortality 1.5 *vs.* 1.25%	In hospital dialysis 0.23 *vs.* 0%
Mitchell 2015 [53]**	ED	Prospective	633	B5	11			One year mortality 17 *vs.* 7%*	One year renal failure 11 *vs.* 2%*
McDonald 2015 [51]**	Patients with GFR 30–60 ml/min (i) GFR < 30 ml/min (ii)	Propensity matched	3278	B2	7.5	8.1	No difference	30 day mortality: 8.9 *vs.* 11% (i) 18 *vs.* 19% (ii)	30 day dialysis: 0.4 *vs.* 0.4% (i) 1.7 *vs.* 0.7% (ii)
				A2	13.1	16.2			
Hemmet 2015 [58]	Hospital	Prospective	600	A4	11	9.5	No difference		
Sonhaye 2015 [17]	ED	Prospective	1292	B5	3.4	1.8	No difference		Renal failure at discharge: 0 *vs.* 0%
Alsafi/2014 [59]	Hospitalized patients aged 70 years or older	Retrospective	1164	B1	9.2	3.5	Higher in CM group		
McDonald 2014 [8]**	Multiple	Propensity matched	21 346	B2	4.8	5.1	No difference	30 day mortality 8 *vs.* 8.2%	30 days dialysis 0.2 *vs.* 0.3%
Davenport 2013 [6]	Hospitalized	Propensity matched	17 652	A1	7	6.9	No difference in patients with GFR ≥30 ml/min Significantly more AKI in patient with GFR < 30 m/min		
Kidoh 2013 [27]	Patients with GFR 15–60 ml/min	Retrospective	470	B6	9.1	8.3	No difference		
Cely 2012 [14]	ICU	Prospective matched	106	C	26.4	35.8	No difference		
Murakami 2012 [28]	Patients with GFR 15–60 ml/min	Retrospective	2034	B3	6.1	6.2	No difference		
Sinert 2012 [19]	ICU	Retrospective	3729	B3	5.7	9	Higher in control group	In hospital mortality 9 *vs.* 6.8%	In hospital dialysis 0 *vs.* 0%
Aulicky 2010 [18]	ICU ischemic stroke and TPA	Retrospective	241	B2	3	3.9	No difference	3 months mortality 18 *vs.* 10%	In hospital dialysis 0 *vs.* 0%
Ng 2010 [54]	ICU oncology patients	Retrospective matched	162	A2	17	17	No difference	In hospital mortality 17.3 *vs.* 21%	In hospital dialysis 2 *vs.* 1%
McGillicuddy 2010 [60]	Trauma	Retrospective	1152	B2	1.9	2.4	No difference		
Lima 2010 [61]	ED	Retrospective	918	B3	5	10	Higher in control group		

CIN: contrast induced nephropathy; CM: contrast medium; CKD: chronic kidney disease.

*Significant difference.

**Overlap in patients included.

[A] AKIN definition 0.3 mg/dl or 50% ↑ in SCr.

[B] KDIGO definition 0.5 mg d/dl or 25% ↑ in SCr.

[C] ↓33% creatinine clearance.

[D] Further deterioration.

Time period: 48 h (2) 24–72 h (3).

## Definition and terminology

When describing or studying renal failure in the setting of CM administration, the label of ‘contrast-induced nephropathy’ was used indiscriminately on all patients. Historically, it was defined as a change in serum creatinine (SCr) that happened shortly after CM administration. This terminology was applied differently in many research endeavors and lead to a wide variety of related but distinct definitions. Moreover, grouping together patients with renal failure after CM into one entity is rather problematic.

On the one hand, the magnitude of change of SCr and the time period over which this occurs varies greatly between studies. Some authors used an absolute change of SCr (as low as 0.3 mg/dl to 0.5 mg/dl) whereas others relied on a percent increase from baseline (25% to 50%) [11]. Similarly, time periods of 24–48, 48–72, 96 h, 5 days, and an entire inpatient stay have been used [11].

On the other, and in our opinion, the more important issue, is the fact that renal failure in the setting on CM consists of not one but two distinct entities: *contrast-induced* nephropathy (CIN) and *contrast-associated* nephropathy (CAN). CIN is defined as a sudden deterioration in renal function occurring within 48 h of CM administration and *after the exclusion of other nephrotoxic factors*. CAN is a general term used to describe *any* deterioration in renal function that occurs within 48 h following intravascular administration of iodinated contrast medium [10,12]. CIN implies causality whereas CAN association of events. The distinction of CIN *vs.* CAN in practice can be difficult, is often unreliable, and most certainly inconsistent between clinicians. Some might go at great length to exclude other causes whereas others might not push investigation quite far [13]. In theory, ‘excluding other causes’ could be interpreted as performing a renal biopsy in some cases. Thankfully, in practice, this is almost never the case.

## Incidence of AKI after intravenous administration

Studies regarding the incidence of AKI after IV CM reported a wide range of frequencies from as low as 2% in some instances [2] to as high as 25–35% [14–16] in others depending on the clinical setting, the type and amount of contrast given, and the definition of AKI. Almost all the studies were done either in hospitalized patients (such as the ICU or after trauma) or in the setting of emergent care (ED, stroke care). Thus, a large portion of the included patients had several risk factors for AKI and many alternative reasons for renal dysfunction (Table 1).

In a prospective single-center study, Sonhaye et al. reported the findings of 1292 patients who were hospitalized after an ED visit and underwent CT imaging. Of those, 620 had CM enhanced imaging, and 672 did not. Three percent of the patients who received CM developed CIN compared to 2% for those who did not have CM. That difference was not significant even after adjusting for other variables [17]. In patients with ischemic strokes, Aulicky et al. found no significant differences in rates of AKI between patients who received CM (3%) or not (3.9%) [18]. A meta-analysis of 28 studies involving 107 335 patients failed to show an increased rate of AKI with CM (OR 0.94 [0.83–1.07]) [11].

Most of the early literature however was done using retrospective studies and compared outcomes of patients that received or did not IV CM. Retrospective trials can be hampered by selection bias. This is undoubtedly the case here as well since clinicians chose different imaging modalities based on the perceived risk for patients. Patients with a presumed higher risk of AKI would have likely received alternative imaging modalities or have their testing postponed or canceled. Indeed, some studies reported rates of AKI that were actually lower in the CM group than in the non-CM group [5,19]. In a large database study on more than 5 million patients, the authors showed that after adjusting for comorbid conditions, the group with CM had 7% *less* AKI [5]. Since CM *cannot reduce* the risk of AKI, the only logical conclusion is that a selection bias is widely present.

Ideally, to overcome these limitations, we should conduct multiple large controlled trials that would prospectively randomize patients to either receive CM or not and monitor the development of AKI. This poses not only tremendous logistical challenges but also such trials would also be ethically difficult to conduct as patients could be randomized to suboptimal studies (such as a VQ scan rather than a CT angiogram for pulmonary embolism) that can jeopardize their care. Thus, researchers have resorted to alternative methods to improve the usefulness of the results from retrospective studies, such as propensity-score matching methods in which patients exposed to CM are matched to others that have a similar risk of getting CM (but did not) based on several factors that are combined and generate a likelihood score (propensity-score).

One of the first such studies by Davenport et al. used a one-to-one propensity-matched matched cohort with multivariate analysis. They included 17 652 patients over a 10-year period at one tertiary care center. Propensity matching was performed using 36 covariates. They defined AKI as a change in SCr over 48 h (an increase of 0.3 mg/dl or 1.5 times above the baseline) and divided the analysis based on the patient’s GFR. Patients with GFR < 30 mL/min/1.73 m^2^ were found to have a significantly higher likelihood of AKI after CM (OR 2.96 [1.22–71.17]) [6].

A study by Mc Donald et al. yielded a different conclusion. Their propensity matching score incorporated 160 ICD-9 codes and six additional clinical variables. There were 12 058 patients admitted over a 10-year period at one tertiary care center. The definition of AKI was an increase of 0.5 mg/dl in SCr over 24–72 h and the analysis was also stratified by GFR [7]. They found that a lower GFR was associated with a higher likelihood of AKI but this was not different in patients who received CM or not. This remained true in patients with GFR < 30 mL/min [7]. Other studies in ED [20] and ICU [21] patients also found no association of CM with AKI even in patients with reduced kidney function. Differences in the matching process, definitions of AKI, and clinical setting may help explain the discrepancies in results in these studies.

If we look closer, however, these studies more likely represent CAN incidence rather than CIN. Almost all of them reported rates of AKI occurring in the setting of CM administration and none actually truly ruled out other causes or entertained alternative explanations. Adding credence to this is a study by Newhouse et al. on 32 161 hospitalized patients [22]. SCr was measured for five consecutive days and patients did not receive any contrast administration. The authors found that more than half the patients had a change of SCr of at least 25%. Patients with creatinine at baseline of 0.6–1 mg/dl, 7% had an increase of 0.6 mg. In those with baseline creatinine over .2.0 mg/dl, there was a 25% increase in SCr. The fluctuation of SCr in this study is similar to the changes used to define AKI in the setting of CM. Since these changes occurred without CM administration, AKI is more likely to be happening in *conjunction with* rather than *due to* CM. We should therefore consider them to represent CAN rather than CIN.

A recent prospective trial yielded similar findings. A cohort of 1009 randomly selected patients from the SCAPIS trial had their SCr monitored over 3 days after receiving intravenous CM. The impact of CM on SCr did not exceed intra-individual background fluctuations [23].

Early molecular markers of AKI were measured in patients with CM and were not found to be elevated. In 501 patients with mild chronic kidney disease, there were no differences in kidney injury molecule-1 (KIM-1) and neutrophil gelatinase-associated lipocalin (N-GAL) in patients that had AKI or not after a CT with CM [24] Also in a study of 77 critically ill patients tissue inhibitor of metalloproteinase 2 (TIMP-2) and insulin-like growth factor-binding protein 7 (IGFBP-7) were not different in patients with or without AKI [25]. All of these substances are considered to be among the early markers of tubular damage. These results further suggest that a substantial proportion (if not all) of the AKI seen in the setting of CM does not represent states of true tubular damage but rather pre-renal hypovolemic (reversible) states. This again supports the notion that reported rates of AKI are more likely to be representative of CAN.

### Risk factors

Decreased baseline renal function is one of the most consistently reported risk factors for the development of AKI. In the setting of intravenous administration of CM, some [6,26] but not all studies [7,20,21,27,28] showed that as well. Although it’s still unclear whether this is due to the hemodynamic changes in hospitalized patients, the underlying risk factors (such as diabetes, hypertension), the patient's medications (such as ACE or ARB), or secondary to the intravenous administration of CM. From our review of the available literature, the risk for patients with GFR > 60 mL/min/1.73 m^2^ seems negligible. For patients with GFR of 30–60 mL/min/1.73 m^2^, the risk does not seem substantial but is probably present (although unclear to which degree) in patients with GFR < 30 mL/min/1.73 m^2^. As a point of emphasis, these conclusions are based on retrospective studies that were not powered to adequately answer that specific question [10]. Another caveat is that appropriate estimation of GFR using SCr-based equations is done with stable laboratory value, this can be difficult to encounter in the hospital setting.

The use of such equations is both practical (readily calculated in most chemistry results) and effective as it applies to the majority of patients with certain exceptions. GFR calculation has limitations in patients with very low (such as in older patients) or high muscle mass; which leads to an over or underestimation of renal function, respectively. An alternative means of GFR calculations in patients can be *via* the use of Cystatin C, which may be more accurate, and less likely to be affected by differences in patient body compositions. A study by Zhang et al. compared SCr and Cystatin C criterion for CIN in regards to predicting adverse outcomes at 12 months; Cystatin C was found to be a more sensitive biomarker than SCr. Unfortunately, due to the lack of widespread availability of Cystatin C testing in the laboratory, this is rarely used in clinical practice [29].

In summary, the results of the available studies show that CIN is likely less frequent than initially thought. Most of the research probably included a substantial number of patients with CAN rather than CIN and the independent risk attributable solely to CM is unclear. Patients with GFR > 60 mL/min/1.73 m^2^ have a negligible risk of AKI. Advanced renal failure (GFR < 30 mL/min/1.73 m^2^) is probably a risk factor for CIN but in the clinical setting, the underlying patient’s conditions and medications also contribute to a significant extent and limit any definite conclusions.

## Differences with intra-arterial contrast administration

In experimental animal models, CM does cause a decrease in renal function. This occurs *via* the induction of vascular disturbances (vasoconstriction and microthrombi), hypoxia of the renal medulla, the release of oxidative species, and apoptosis [30]. Whether animal models can reliably replicate human physiology is an unresolved debate but it seems that there is agreement that CM does affect the kidney.

CIN after IA is thought by some to be a frequent cause of AKI in hospitalized patients [31]. So why does CM administration would cause more often AKI in the arterial setting than in the venous one? Few possible reasons have been proposed.

One explanation raised by some is that the volume of CM is higher in IA than IV. While contrast volume during a routing coronary angiogram can be as little as 50–60 mL, it can rise quickly when a procedure is performed. By comparison, the CM amount in CT scan can vary from 100 to 150 mL (depending on the patient’s weight and study). When contrast is expressed by grams of iodine/GFR, rates of AKI seem to be similar between IA and IV [32].

Another explanation is that CM presents to the kidney in higher concentration after IA (because it is less diluted). This *first pass exposure* happens during ventriculography, or when the injection of CM is done directly in the renal arteries or the portion of the aorta above them [33]. A *second pass exposure* (more diluted) occurs when CM is injected into the venous system, the right heart, or in the aorta below the renal arteries. In the case of coronary injection, the CM drains into the venous system first and then presents to the kidneys with a lower concentration [34]. This explanation is also not satisfying.

Three other explanations can help explain the discrepancy in our opinion. First, it is possible that CIN in IA is being overestimated as well. High-osmolality CM was utilized in many of the initial studies. As discussed above, CM can cause high rates of AKI owing to its extremely elevated concentration. It is possible that this belief remains engrained in clinicians’ minds because of those initial studies despite the new more reassuring data [4]. This was described by Davenport as ‘…incomplete penetrance of new knowledge into scientific practice, latent bias related to historical precedent …’ [10].

Also, when looking at elective percutaneous coronary intervention (PCI), the AKI rates (1–2%) are much lower than those occurring during ST-elevation myocardial infarction (STEMI) (10–20%) [9]. This suggests that hemodynamic changes also play a role in the development of AKI with IA. In a study of 931 propensity-matched pairs of patients with STEMI, Caspi showed that AKI rates were not significantly different with and without PCI (8.6 *vs.* 10.9%, *p* = 0.12). The occurrence of AKI was mainly due to older age, baseline renal dysfunction, heart failure, and hemodynamic instability [35]. The findings from that study suggest that the CIN cases that occur are probably balanced by a reduction in other AKI cases due to the improvement in hemodynamics with aggressive earlier management of the STEMI. It is possible that, when authors discuss the high incidence of CIN after IA, they are in fact they are referring to renal failure that occurs at a time of high hemodynamic instability in patients with many risk factors for AKI.

Finally, it is possible that some proportion of AKI seen after contrast that is labeled CIN is in fact due to cholesterol emboli. Accessing the arterial system, moving catheters inside the vessels, and dilating lesions can cause debris to migrate and damage the kidneys [36]. Clinicians think of cholesterol emboli mainly when AKI occurs with other systemic findings, such as livedo reticularis. This is likely an underestimation since a definitive diagnosis can only be ascertained by performing renal biopsies on patients' CIN. This rarely (if ever) happens in patients with AKI after PCI.

In summary, CM does cause renal dysfunction in animal models. CIN after IA is estimated by some to have a significant incidence. The reasons for differences between rates of CIN after IV or IA include long-held beliefs that stem from findings of high-osmolar CM, the clinical setting of STEMI, and under-recognized rates of cholesterol emboli.

## Prevention of CIN

The efficacy of specific preventive methods or therapies for AKI in the setting of CM remains unclear. Ideally, we would identify high-risk patients and use an agent with a good safety profile that would significantly reduce the incidence of AKI. Stratifying patients into risk categories by GFR is a reasonable strategy. Patients with GFR > 60 mL/min/1.73 m^2^ should not be considered at risk, whereas those with GFR < 30 mL/min/1.73 m^2^ should be. For the patients with GFR 30–60 mL/min/1.73 m^2^, there is no specific high-quality evidence that can guide our management and an individualized approach should be done. The *ACR/NKF* guideline recommends considering giving fluids to patients with GFR 30–45 mL/min/1.73 m^2^ but not those 45 mL/min/1.73 m^2^ or greater [10].

Modalities that have been studied for CIN prevention include IV crystalloid hydration with normal saline, sodium bicarbonate, or *N*-acetylcysteine (NAC) to name a few. As stated in the introduction, the overwhelming majority of the studies were performed with IA CM administration with very few focusing on IV CM specifically.

NAC was one of the most studied agents owing to its low cost, good safety profile, and potential to scavenge free radicals (one of the mechanisms of CIN). Randomized controlled trials with IA failed to reach definitive conclusions regarding the potential benefit of NAC with some studies showing small treatment effects whereas others did not [37]. A meta-analysis by Xu analyzed (among others) findings from seven studies looking at only 867 patients that had CT scans with contrast. By comparison, in the same paper, 9399 patients had coronary angiograms [38]. The authors found that the incidence of AKI in the NAC group was 7.7% compared to 14.8% in patients without NAC (RR: 0.51, 95% CI: 0.29–0.89, *p* = 0.02) in the subgroup that got CT scans.

Hydration with an intravenous crystalloid solution either sodium bicarbonate or normal saline has also been extensively studied. There is, unfortunately, no robust evidence pointing at a definite benefit of a specific IV fluid regimen over another. What is clear however is that avoidance (and correction) of hypovolemia is critical [39]. Most of the studies have compared one regimen of hydration *vs.* another and very few looked at IV crystalloid *vs.* not in high-risk patients. One such study analyzed the impact of 1 h hydration with sodium bicarbonate *vs.* no hydration in patients with CKD (GFR < 60 mL/min/1.73 m^2^) suspected of having an acute PE. There were 138 patients included in the intent-to-treat analysis and the authors did not find a statistically significant difference between the groups. Of note that study enrolled very few patients with GFR < 30 mL/min/1.73 m^2^ [40]. One study on a subgroup of patients with GFR 30–60 mL/min/1.73 m^2^ with IA CM showed that no hydration was non-inferior to IV saline (0.9%) [41]. Since no specific regimen has been shown to be superior to the other, the timing and rate administration should be individualized based on the urgency of the procedure and the volume status of the patient. Generally, preference would be for normal saline (0.9% NaCl since it is readily available) administration over several hours around the CM timing [10].

Some studies have looked at combining NAC and with different IV fluids regimens for the prevention of CIN in patient receiving IV CM. In one randomized double-blinded study, Traub compared giving NAC plus normal saline with normal saline alone. 357 completed the trial, and there were no differences between groups (7.6 *vs.* 7.0%). There was, however, a significant difference in AKI rates in patients receiving less or more than 1 liter of fluids: 12.9 *vs.* 3.3%. The Odds ratio was 0.41; 95% confidence interval 0.21–0.80 per liter of intravenous fluids [42]. A study by Kama looked at whether adding NAC or sodium bicarbonate to normal saline was equivalent to normal saline alone [43]. There was no difference in AKI rates between groups.

In addition, diuretics should be avoided in the period surrounding CM administration unless the patients are in congestive heart failure. Other potentially nephrotoxic medications (such as NSAIDs) should also be withheld. For patients on treatment with medications that affect the renin-angiotensin-aldosterone system (RAAS), clinicians should also stop the medications. In a meta-analysis of 12 studies [44], Jo et al. found that in patients treated with these agents chronically, withholding them lessened the likelihood of AKI. There was also a hazard of continuation in older patients and patients with chronic kidney disease [44]. Stopping these agents would avoid hypotension and allow the renal system to better auto-regulate. This is of even greater importance in patients hospitalized with acute illness.

**Figure 1.**
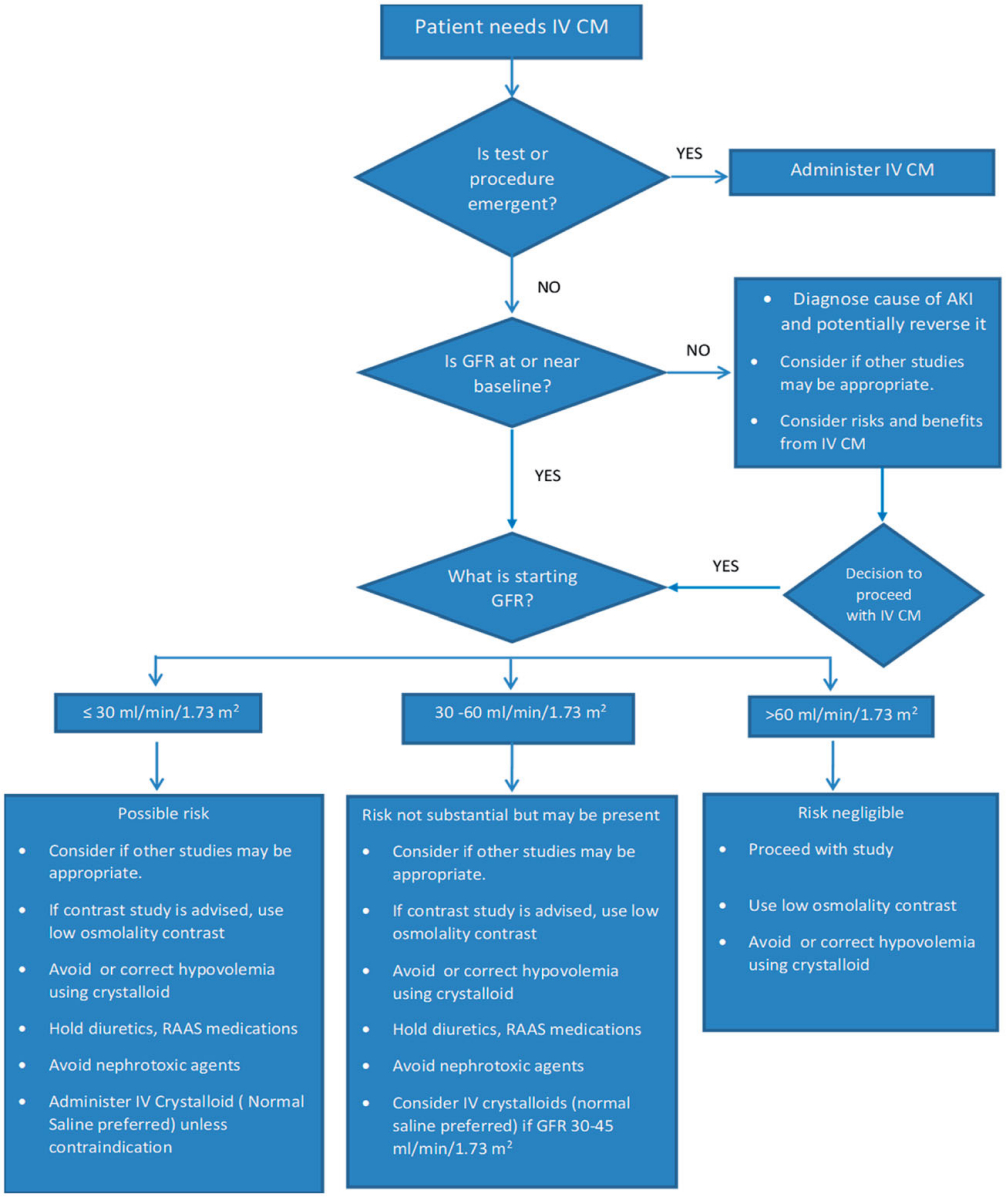
Suggested algorithm for clinical decision-making using intravenous contrast. Timing and rate of administration of crystalloids should be individualized based on the urgency of the procedure and the volume status of the patient.

In summary, when treating patients that are about to get CM enhanced CT, prophylactic intravenous administration of crystalloid should be administered to high-risk patients, such as those with GFR < 30 mL/min/1.73 m^2^ and possibly to those with GFR 30–45 mL/min/1.73 m^2^ [10,45]. For the latter category, the benefits of hydration should be balanced with the potential risks of volume overload and heart failure. The clinician should use their judgment as to the time period for administration and whether delaying a study for some time while giving IV fluids would be beneficial. As far as prophylaxis with NAC in addition to IV fluids, there does not seem to be a benefit. Diuretics, nephrotoxic medications, and medications affecting the RAAS system should be withheld around the time of the study unless other more compelling indications supersede stopping them (Figure 1).

## Consequences of CIN after intravenous administration

The true incidence of CIN remains contested. Therefore it makes sense that we should also scrutinize the consequences of IV CM administration. In general terms, AKI episodes are associated with a higher incidence of chronic kidney disease and mortality. This association however does not of course prove causality. Adverse events occurring shortly after CM administration can be considered possibly linked whereas those that happen long after are likely related to underlying risk factors. Let us take for example a patient with diabetes, hypertension, and coronary artery disease who develops CIN that completely resolves in few days but then suffers from an end-stage renal disease requiring dialysis (ESRD) 3 years later and subsequently passes away. Can we reliably link the episode of CIN to the ESRD? Maybe. What if the ESRD occurred 5 years later? Or if the patient passed away 10 years later? Can we still say that a self-limited CIN episode resulted in death a decade later? This is the kind of challenge that researchers face when they have to conclude regarding CIN.

For outcomes to be clearly linked to an event, there needs to be a clear temporal sequence and a plausible pathophysiologic connection. Important outcomes of interest that meet both these conditions would be the development of chronic kidney disease, the onset of ESRD, and mortality [46]. Potential consequences can be divided into short, medium, and long-term. Short-term outcomes are clearly related to CIN and would happen as a continuation of the disease episode up to 1 month. Medium (after 1 month) and long-term consequences (after 1 year) are thought to occur later [47].

The majority of published studies showed that mortality was no different in patients who receive CM [8,15,18,19,48–54] (Table 1). In-hospital dialysis following IV CM was extremely low in most studies except for reports that analyzed patients with sepsis [8,15,17–20,48–51,53–55] (Table 1). Thus, the short-term outcomes seem to be favorable in the overwhelming majority of cases. For longer-term follow-up, studies that looked specifically at IV CIN show, that mortality is increased but not dialysis [20,53]. A study from the Taiwan health system examined 7100 propensity-matched patients and evaluated for CM results in more dialysis needs. Patients with IV CM did not have a higher risk for dialysis overall [56]. When the authors analyzed patients that received more than one exposure to CM/year, however, the risk of ESRD increased significantly. One caveat is that the necessity of repeated exposure suggest that those patients have other underlying reason that could themselves account for the worse outcomes.

As discussed before, it is possible that the incidence of CIN differs after IA or IV. Once CIN occurs, however, there is no good reason to think that its outcomes would be different depending on the mode of administration of CM. However, in the literature, we can see clear differences. In studies about CIN after IA CM, this entity is clearly associated with higher mortality, more dialysis, and CKD.

So how do we reconcile these findings? One way to look at it is that comorbid conditions, risk factors, and the clinical setting for IA administration can explain these differences. We favor this explanation since many of the cases labeled CIN are in fact CAN and the reported outcomes actually reflect events happening *around the time* of CM administration rather than *due to* CM administration. Another problem with research studies is that they lump together patients with similar conditions and risk factors that have various degrees of severity. If we take two patients with the label diabetes, for example, one could have had diabetes for 10 years and be uncontrolled whereas the other can have diabetes for 2 years and be doing quite well. Does it mean that both patients have the same risk of bad outcomes? Common sense says no, but for ease of conducting research, authors have to classify patients into the same category so the intrinsic severity of the individual disease is often hidden.

In addition, it is likely that selection bias precluded sicker patients from getting IV CM in the aforementioned studies. Thus, it is conceivable that in some high-risk patients CIN might not be harmless. However, this explanation still does not resolve the dilemma we face: are the outcomes due to CIN itself or surrounding factors. In our opinion, we think that CIN may at least serve as a signal that patients are at high risk for adverse events but whether it definitely impacts outcomes is unclear.

In summary, it is difficult to clearly link events that happen long after CIN to it. It seems that short-term consequences of CIN are generally favorable, whereas the observed adverse long-term consequences might be more reflective of underlying risk factors and conditions. Studies showed differences in outcomes after CM for IV and IA. The worse outcomes are seen after IA is likely reflective of conditions and events that happened around the time of the administration of CM.

## Conclusion

For many years, CIN after IV administration was thought to be of common occurrence and to have dire consequences. Previous research efforts were hampered by the heterogeneity of the definition of CIN and by not distinguishing properly CAN from CIN. Recent studies showed that its incidence is likely much lower than previously thought and its outcomes were favorable and more related to underlying risk factors. Advanced renal failure (GFR < 30 mL/min/1.73 m^2^) is likely a risk factor for CIN genesis, and those patients should receive IV fluids as a preventative measure around the time of CM-enhanced CT scans. Clinicians should take into account these findings when deciding on how to care for their patients. Renal failure itself should not serve as an absolute contraindication for receipt of CM and a careful risk/benefit analysis should be done on a case-by-case basis to decide on the optimal course of management.
